# Assessing the Potential of Low-Cost 3D Cameras for the Rapid Measurement of Plant Woody Structure

**DOI:** 10.3390/s131216216

**Published:** 2013-11-27

**Authors:** Charles A Nock, Olivier Taugourdeau, Sylvain Delagrange, Christian Messier

**Affiliations:** 1 Departement des Sciences Biologiques, Université du Québec à Montréal, Montréal, QC H3C 3P8, Canada; E-Mail: o.taugourdeau@gmail.com; 2 Departement of Natural Sciences, University of Quebec in Outaouais (UQO), 58 Main Street, Ripon, QC J0V 1W0, Canada; E-Mails: sylvain.delagrange@uqo.ca (S.D.); christian.messier@uqo.ca (C.M.)

**Keywords:** 3D plant measurement, Microsoft Kinect, Asus Xtion Pro Live, stem diameter, branch diameter, 3D camera, canopy structure, point clouds

## Abstract

Detailed 3D plant architectural data have numerous applications in plant science, but many existing approaches for 3D data collection are time-consuming and/or require costly equipment. Recently, there has been rapid growth in the availability of low-cost, 3D cameras and related open source software applications. 3D cameras may provide measurements of key components of plant architecture such as stem diameters and lengths, however, few tests of 3D cameras for the measurement of plant architecture have been conducted. Here, we measured *Salix* branch segments ranging from 2–13 mm in diameter with an Asus Xtion camera to quantify the limits and accuracy of branch diameter measurement with a 3D camera. By scanning at a variety of distances we also quantified the effect of scanning distance. In addition, we also test the sensitivity of the program KinFu for continuous 3D object scanning and modeling as well as other similar software to accurately record stem diameters and capture plant form (<3 m in height). Given its ability to accurately capture the diameter of branches >6 mm, Asus Xtion may provide a novel method for the collection of 3D data on the branching architecture of woody plants. Improvements in camera measurement accuracy and available software are likely to further improve the utility of 3D cameras for plant sciences in the future.

## 1. Introduction

Understanding the structure and characteristics of plant canopies is crucial for developing a more thorough understanding of many facets of plant ecology. The study of above ground plant architecture provides insights into differences in plant resource capture strategies and specific functional acclimation or adaptation to a varying environment [1], but also allows quantification of the aboveground biomass and in turn the carbon stored in terrestrial ecosystems [2–4]. Finally, detailed 3D data informs the study and simulation of spatio-temporal aspects of plant development, physiology and growth [5].

The inclusion of accurate descriptions of canopy architecture and development into plant modeling has led to the development of an important field of research in plant science—functional-structural plant modeling (FSPM) [5]. FSPM presently offers new insights in simulating-analyzing plant architecture in relation to genetic variability or in response to experimental manipulation and then evaluating the implications of theses sources of variations on plant performance, crop production, pathogens invasion, and soil stability [5,6]. FSPM approaches are constraints by the quality of plant architectural measurements because 3D plant growth models calibrations require a wealth of architectural data in order to accurately simulate tree development and growth *in silico* [7–9]. For example, to compute carbon assimilation using simulations of light interception by canopies, researchers require accurate data on branches and leaves and their orientation in 3D space [10].

Methods for measuring the structure of plant canopies vary greatly in the scale addressed as well as the complexity of the data generated. At larger scales (*i.e.*, tree to stand), tree canopies can be characterized airborne LIDAR [3]. At finer scales (*i.e.*, branches to several trees), ground-based measurements can be performed using Terrestrial Light detection and ranging Scanning (TLS) [2,4], 3D plant digitizers [11–13], image based systems [14–17], or by using destructive as well as non-destructive manual methods [18,19]. In general, measurements for FSPM inputs have been derived from ground-based measurements.

Despite the importance of detailed 3D models in understanding many aspects of plant ecology, acquiring the necessary data to parameterize models has remained challenging for plant scientists, both due to the cost and complexity of the instruments/methods required and to the amount of time required. For example, detailed information on the woody structure and foliar distribution in plant canopies can be generated using 3D magnetic digitizers [11–13], but this method can be very slow (e.g., up to two days for a ∼2 m tall *Betula alleghaniensis*) [11]. Measurement rapidity is thus a key reason why the number of individuals and species measured in 3D remains small relative to woody plant terrestrial diversity [11,13]. An approach that has been used with success is TLS, which is well suited to capture 3D architecture [2,4,20–22], but the high cost has generally prohibited the regular use and application of TLS. In addition, the proprietary nature may limit flexibility in modifying existing software to allow for different or additional measurements to be taken from point clouds. Finally, manual measurements of plant canopy structure from foliage or branch line-intercept methods [18], or from destructive harvests of foliage and branches [19], can provide detailed information but the time required to acquire and adequately post-treat the information limits replication across individuals and species. Thus, the search for instruments that are both cost effective, facilitate rapid measurement and are sensitive enough to generate meaningful data continues.

Video cameras capable of sensing depth have existed for some time, but the release of the Kinect sensor [23], has made such sensors much more accessible due to the low-cost and quality of the depth sensing. This type of low-cost 3D depth camera builds a depth image by emitting a structured pattern of light and by measuring the deformation of the structured lighting pattern that is caused by structures in the scene [24]. While initially developed explicitly to track body position and movement for video games, its ability to capture detailed point clouds from environments has resulted in a rapid increase in popularity with researchers in a diverse array of fields ranging from geology to artificial intelligence. Surprisingly, few researchers have examined the utility of 3D cameras for measuring plant traits (but see [25,26]), and we are not aware of studies examining the potential application of 3D cameras to measure woody plant architecture.

3D cameras such as the Kinect and Xtion have been developed around the flexible open source SDK and libraries (e.g., OpenNI, OpenCV, Point Cloud Library in C++), which has facilitated a rapid expansion of software tools and packages. For example, the nature of 3D video presents a novel opportunity to conduct 3D object scanning and modeling in real time (in contrast to static acquisitions from multiple viewpoints and subsequent scan registration). Open source software implementations for continuous 3D scanning are now widely available and may present a novel opportunity for fast and cost-effective capture of plant 3D architecture, while also potentially providing a useful alternative approach to dealing with occlusion (normally multiple static acquisitions from different angles are taken). However, it remains uncertain how sensitive 3D cameras are with respect to measuring the geometry and the aerial structure of plants (e.g., branch diameters), or how well continuous 3D scanning software functions for plant scanning. Therefore, their suitability as a tool for capturing plant architecture remains uncertain.

In this paper our overall goal was to explore the utility of 3D cameras (e.g., Microsoft Kinect and Asus Xtion Pro Live) to measure plant canopy architecture by determining the detection limit and accuracy of measurement for branches varying in diameter. Specifically, using a series of images captured with an Asus Xtion Pro Live we asked: (1) what is the minimum measurable branch diameter; (2) how accurate are estimates of branch diameter from point clouds; and (3) what is the effect of varying the distance between the camera and the subject being measured? Finally, we also tested existing continuous 3D scanning applications KinFu, ReconstructMe, and Skanect for their ability to detect and quantify woody branches and to determine the correspondence of measurements with static acquisitions.

## 2. Methods

### 2.1. Structured Light 3D Cameras

The Asus Xtion Pro Live is a depth camera system comprised of a camera dedicated to RGB imaging, a second camera dedicated to IR detection and an infrared structured light source. The depth resolution is ∼10 mm at close range, but this resolution varies with distance from the sensor [24]. The characteristics of the Asus Xtion Pro Live and a popular competitor—Microsoft Kinect—are summarized in Table 1. The cameras are very similar and are both based on PrimeSense technology [27].

### 2.2. Apparatus, Point Cloud Capture and Measurement of Branch Diameters

#### 2.2.1. Branch Holding Apparatus

**Figure 1.**
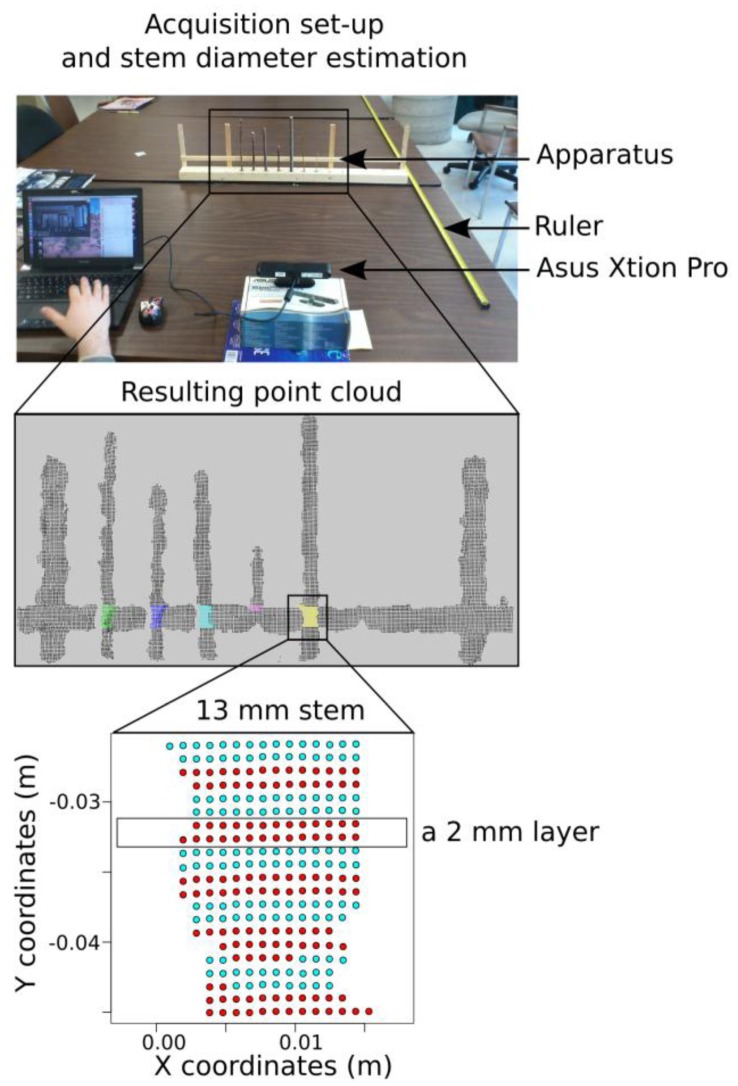
Illustration of the experimental method for quantifying the threshold for diameter detection and the change in accuracy of diameter measurement with distance from static acquisitions with a 3D camera.

We chose seven straight branches of *Salix sp.* ranging in diameter from 2 mm to 13 mm and affixed them to a wooden board (Figure 1). The segments were evenly spaced (50 mm) and oriented vertically (Figure 1). True branch diameters were measured using an electronic caliper at a reference height that was indicated by a straight horizontal wood segment placed ∼5 cm behind the branches. Branch diameters were chosen to reflect a range of possible diameters at the threshold of detection of the 3D camera, including branches that were known to be much smaller than the smallest detectable diameter (2 and 3 mm, based on preliminary tests).

#### 2.2.2. Static Acquisitions: Point Cloud Capture

To test the effect of distance from the sensor on the measurement accuracy and detection limit of the sensor, the branch holding apparatus was placed at the end of a long table in the lab facing the Xtion sensor. Starting at a distance of 2.5 m, data was captured (10 images) and then the apparatus was moved 0.25 m closer to the camera (while keeping the apparatus in the center of the cameras view) until the distance between the camera and the apparatus was 0.25 m. The experiment was conducted in a lab with lighting primarily from fluorescent fixtures, some natural daylight from windows, but no direct sunlight.

Based on available source code from PCL for requesting data streams from OpenNI compatible cameras [28], a dedicated program that captures the point cloud from the 3D camera was compiled. At each distance, using a laptop (Toshiba Tecra R840-021 running Ubuntu v.12.04), 10 data captures of the scene were saved as a point cloud file for subsequent processing (.pcd: ascii point cloud data file format). The 10 repetitions were performed to assess the variability of the camera output for virtually constant conditions. A laptop was used to mimic field constraints (e.g., requirements for compactness and wireless operation).

Stem diameters were measured from the point cloud files using an automated procedure: for each branch, a 2 cm stem section around the reference height (cf. 2.2.1), subdivided into 10 horizontal layers, was extracted from each point cloud data files (Figure 1). For each 2 mm layer, the stem diameter was estimated by the point cloud range on the x-axis:

(1) $$D_{1}=\text{Max}(X_{1})-\text{Min}(X_{1})$$

with D_l_ the estimated stem diameter of the layer l, and X_l_ the coordinates of the points of the corresponding layer. The branch diameter was assumed to be the mean of stem diameters per layer. Each of the 10 images was analyzed separately to assess the measurement variability.

Data are presented for the branch x distance cases where the branch is properly captured for the 10 images (*i.e.*, the height of extracted point data are closed to the height of the extraction window: 2 cm). This filtering is made to highlight measurement precision when the branch is always captured by the 3D camera.

#### 2.2.3. Estimates of Stem Diameters from Continuous 3D Scanning

In addition to static acquisitions we also tested the detection ability and accuracy of three popular 3D object scanning and modeling implementations: (1) KinFu from PCL; (2) ReconstructMe; and, (3) Skanect. Differences in the programs are summarized in Table 2.

KinFu reconstructs a single dense surface model with smooth surfaces by integrating in real time the depth data from the 3D camera over time from multiple viewpoints. As the sensor is moved, the depth data reference system slightly shifts. KinFu uses a real-time registration algorithm to estimate this shift and transpose the new frame into the scene reference system to comprise a single reconstruction of the objects or environment of interest. KinFu is available within the Point Cloud Library [29], and aims at providing an open source implementation of Kinect Fusion [30]. We compiled KinFu from the source code [31], without modifications to the code or the default settings. We assume that ReconstructMe and Skanect are similar in their approach to 3D scanning.

ReconstructMe is available as a free download, although the software is commercial and hence there are limits to the software functionality to encourage users to purchase a license [32]. The first of these limits is a waiting period of 20 s during file writing, while the second (and more problematic) is that spheres are added at random locations to any saved mesh file. For our purposes, the addition of the spheres did not interfere with our ability to estimate the diameters of the small branch segments and thus the detection limits of the software. The different settings included head high detail, standing human high detail, 1 m^3^ high detail, and 3 m^3^ high detail.

Skanect is available as a free download but requires a license to be purchased for full functionality [33]. Until a license is purchased, the resolution of exported mesh files is limited (Table 2). Despite this limitation, we still tried to qualitatively assess the potential of Skanect for plant architectural measurement based on the full resolution mesh that is viewable, but not exportable. Similar to ReconstructMe, one can select the scale at which you plan to scan in Skanect in order to optimize settings for the type of scanning. Possible Skanect settings were: body, object, half a room, and room.

For all of the 3D object scanning and modeling implementations tested we attempted scanning from ∼60 cm and ∼100 cm away from the branch apparatus and moved through an arc of approximately 120 degrees (60 degrees on each side of the center). We did so for using different detail settings if the implementation provided the option (Table 2).

At both 60 cm and 100 cm distances, we attempted to generate 5 replicates of the mesh of the scene. If successful (*i.e.*, mesh objects for branches were generally cylindrical), we saved each replicate as a .ply file (binary mesh file format). We then used the linear measurement tool in Meshlab [34] to quantify the diameter of each of the branches present. The diameter was measured at the same height that was used in the static acquisitions and from straight on.

Protocol differences between static and continuous 3D acquisitions are due to intrinsic differences between the two approaches: for continuous scanning and modeling, an acquisition is already the mean of a large number of repetitions (frame per sec * acquisition duration), and the distance between the camera and the apparatus is an average as the apparatus is displaced during the acquisition.

Continuous 3D object scanning and modeling tests utilized an Intel based desktop with 8 cores (3.4 Ghz per core) and 16 GB RAM running Windows 7 Professional, as well as a CUDA equipped graphics card—we used a Nvidia Geforce gtx 650 with 1 GB RAM and 384 CUDA cores.

#### 2.2.4. Testing 3D Scanning on Real Plants

We also conducted 3D scanning on two real plants. The first was a section of the branching structure of a large mature *Acer platanoides* that was 24 mm in diameter at the base and 1 m in length; the second was a large cactus-like euphorbia approximately 1.5 m tall. Two acquisitions protocols were tested: (1) Continuous scanning with KinFu software from 1 m distance; and (2) Static scanning from 5 different points of view (∼1 m distance). CloudCompare was used for the registration on theses static scans [35]. For the maple branch, 41 segments widths were manually recorded on the branch (min: 2.9 mm; max: 24.2 mm) to have base-line data to characterize the detection limits of the two approaches (continuous registration *vs.* static scanning).

## 3. Results

### 3.1. Static Acquisitions and Detection Limits

**Figure 2.**
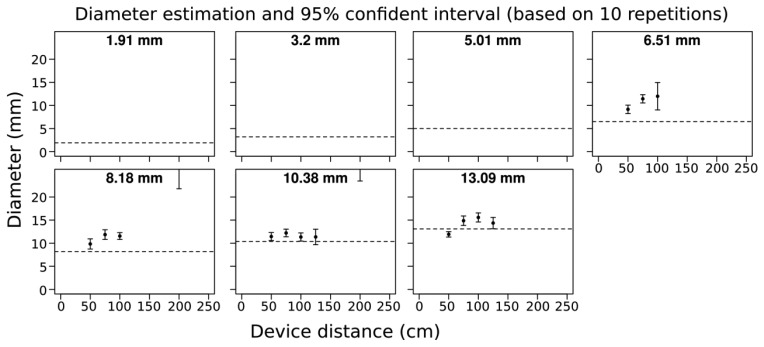
Threshold for diameter detection and accuracy of diameter measurement as a function of distance from branches of *Salix* of various diameters (shown in bold at center, top of each panel) measured from 3D images captured using the Asus Xtion Pro Live. Whiskers show 95% confidence intervals ^†^ for the mean, calculated from 10 replicate acquisitions.

We found that branches below 6.5 mm in diameter were not detected by the camera *i.e.*, there was no point cloud data generated. For branches ∼7 mm in diameter bias was +4 mm, and for branches greater than 7 mm in diameter measurement bias decreased to +2 mm. We found that the accuracy was similar from 0.5 m up until a distance of 1.25 m from the target for all detected branches (Figure 2). Overall, errors were related to overestimation of diameter and the degree of overestimation was generally consistent. Variability among the 10 image captures was also quite small within the optimal 1.5 m scanning distance. Beyond 1.5 m distance, the acquisition quality was quite poor, and in most cases the acquisitions were not analyzed because the branch elements were not well captured (see Method part for details).

### 3.2. Continuous 3D Scanning of Branches

**Figure 3.**
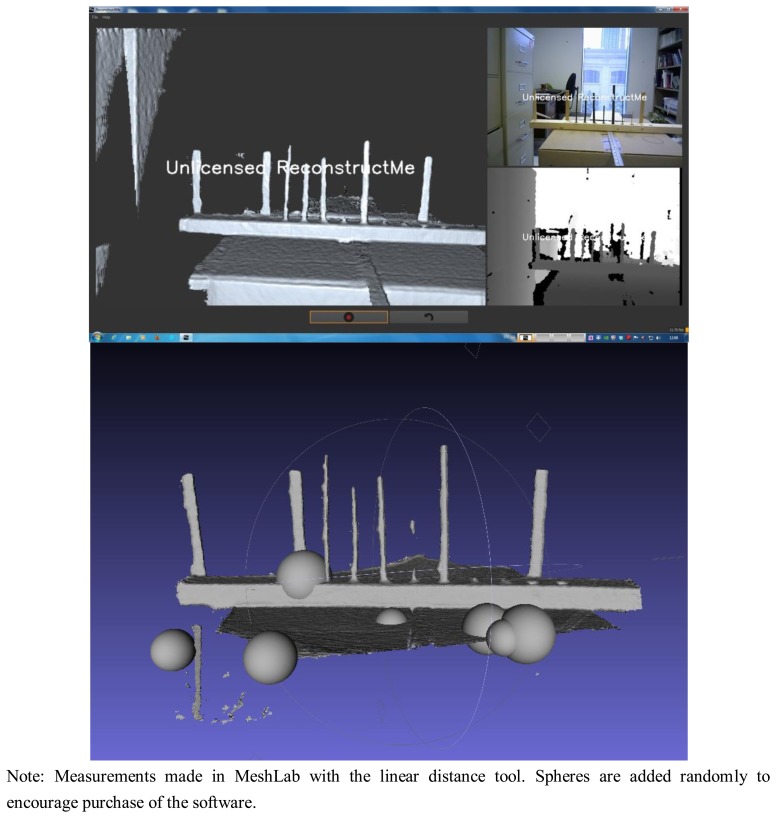
Top: screen capture of ReconstructMe showing the generated mesh (left), the RGB camera output (top right) and the depth camera output (bottom right). Bottom: resulting mesh from ReconstructME viewed in MeshLab. Spheres are added to generated meshes if a license has not been purchased. From left to right starting at the second vertical object: 7, 8, 10, 5, 13, 3 and 2 mm.

**Figure 4.**
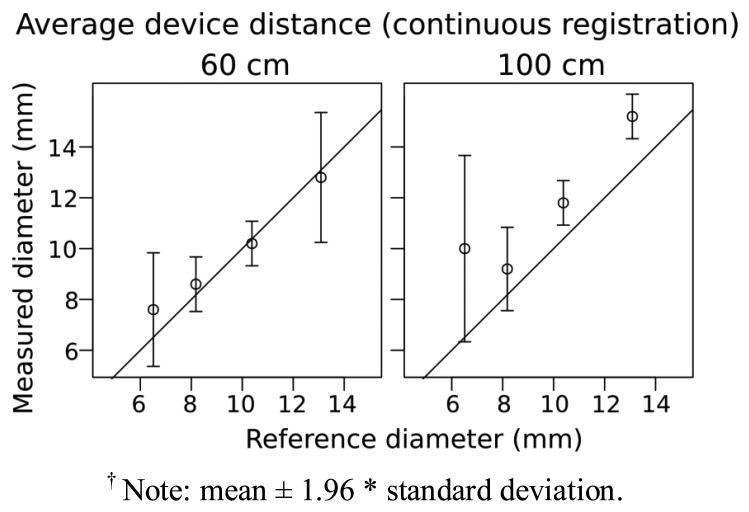
Comparison of reference and measured values for branches varying in diameter. Measured values were obtained from mesh generated using ReconstructMe and an Asus Xtion at 60 cm and 100 cm (other software tested did not yield measurable meshes). Circles show the mean of 5 scans and whiskers show 95% confidence intervals ^†^ for the mean; 1:1 relationship between reference and measured values indicated with a solid line.

For ReconstructMe we attempted to use head high detail mode but latency issues and a low number of frames per second (fps) (fps, <3–4) prevented us from generating meshes of the branches despite having a CUDA equipped graphics card. For the other modes—standing human, 1 m^3^ volume and 3 m^3^ volume—the scanning was much faster at 15–25 fps. For the 5 mm diameter stem there was some indication that it was captured, but the geometry was poorly defined (Figure 3). Branches <5 mm were not at all visible. For the 1 m^3^ volume high detail setting results were quite similar to the standing human high detail setting except that 5 mm was more clearly captured, and the overall shapes were better approximated as cylinders. Using the “1 m^3^ high detail” setting, measurements for diameter of the 7, 8, 10, 13 mm branches were within 1–2 mm when scanned at a distance of ∼60 cm (Figure 4). At a greater distance (1 m), the accuracy of diameter measurement declined by ∼2 mm (Figure 4).

**Figure 5.**
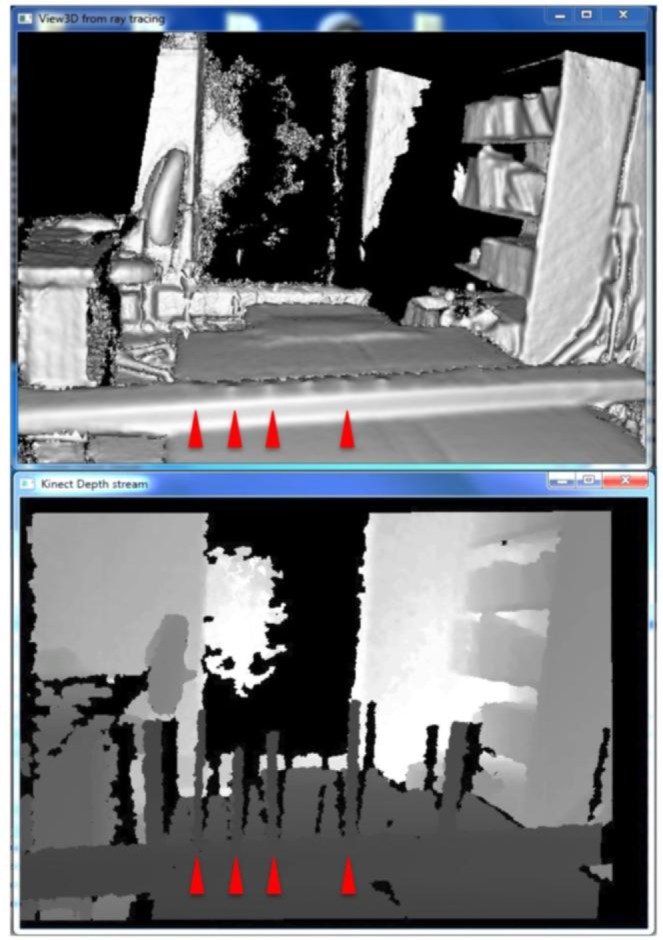
Example of depth stream (top) and resulting mesh (bottom) from the software KinFu and an Asus Xtion 3D camera. Notice that despite the presence of the branches in the depth stream that do not appear in the resulting mesh—suggesting that “filtering” of features of the generated mesh and their tenability will be important for continuous 3D plant scanning. Scanning time was <1 min.

When scanning with KinFu, low fps values were not an issue (c. 20 fps). However, at either the 60 cm distance or the 1 m distance the branches would appear initially in the generated mesh, but within a timeframe of 20 s they would disappear (Figure 5). We thus did not save the results of our attempts to generate mesh for the branches with KinFu. Interestingly, from the window displaying the depth stream it was clear that there was information for the branches in the data stream (Figure 5).

**Figure 6.**
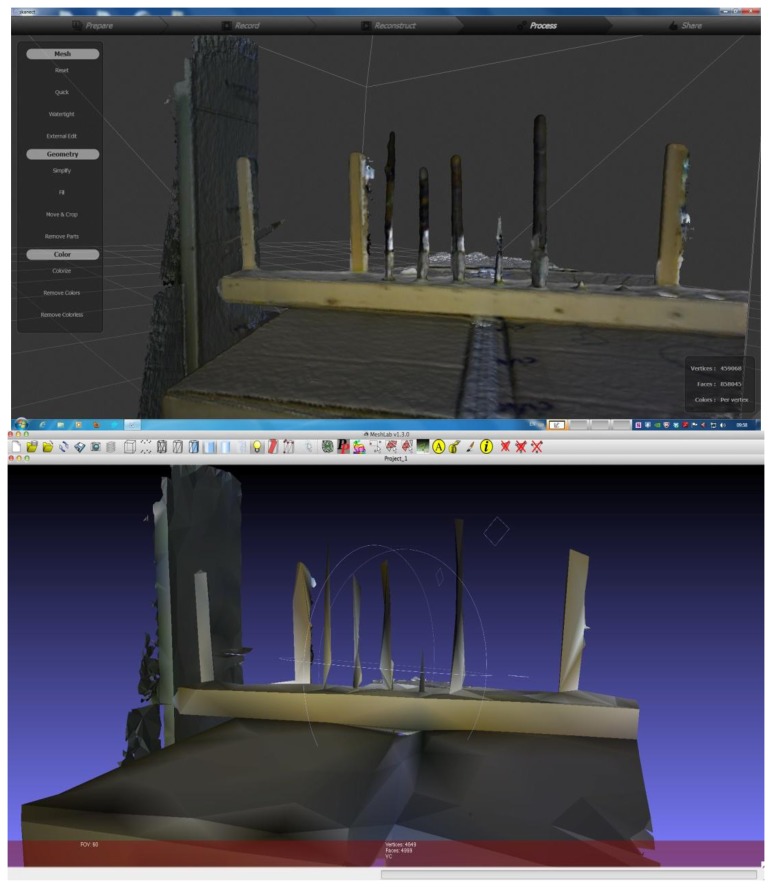
Example of 3D scanning of woody branches using Skanect and an Asus Xtion. Top: original high-resolution mesh of woody branches scanned from ∼60 cm using Skanect. Bottom: a low-resolution mesh export is the only option available until a license is purchased.

With Skanect, scanning was inhibited by low frame rates when set to the highest detail mode (body, fps < 3–4); similar to scanning at small scales and in high detail mode with ReconstructMe. Although it was not possible to quantitatively compare the accuracy of diameter estimation from Skanect due to the limitations on the resolution of the exported mesh (Figure 6), we qualitatively determined that the measurement performance was quite similar to ReconstructMe (Figure 6).

### 3.3. 3D Scanning of Real Plants

**Figure 7.**
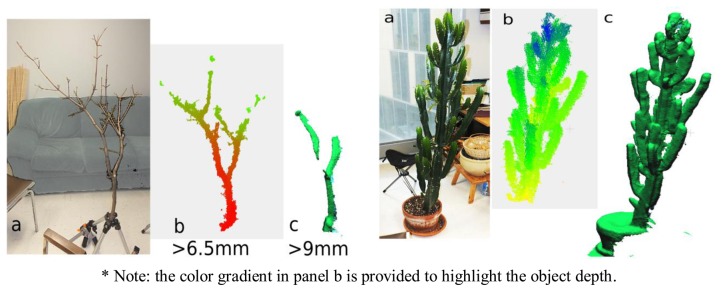
Examples of 3D scanning of real plants. Maple branching system at left, and cactus-like euphorbia tree at right: (**a**) photograph; (**b**) point cloud based on 5 merged static scans *; (**c**) wire-frame meshes obtain by the continuous 3D scanning (KinFu software).

For the maple branch, a number of the smaller diameter branch elements were not captured in the scan. More precisely, of the 41 segments widths for which data were manually recorded on the branch (min: 3 mm; max: 24 mm), those segments with diameters below 6.5 mm were not detected by either static image acquisitions or by 3D scanning with KinFu. For segments between 7 and 9 mm, only static scanning resulted in segment detection, whereas segments ∼10 mm and larger were detected by both methods (we did not have segments >9 mm but <10 mm in diameter). The cactus like euphorbia was well acquired thanks to the width of it axes (Figure 7).

## 4. Discussion

### 4.1. Discussion

Combined with a rapidly expanding open-source code-base (e.g., Point Cloud Library), 3D cameras such as the Asus Xtion Pro Live^©^ can produce valuable data on the woody structure of plants. While previous studies have explored the technical limits for the sensor [23], and others have measured plant attributes such as crown volume [25] or leaf area [24] using 3D cameras, this is the first study that can inform plant scientists considering using 3D cameras for the measurement of woody plant stems in 3D.

#### 4.1.1. Determining 3D Cameras Detection Limit and Accuracy of Diameter Measurement for Branches

We sought to determine the minimum measurable branch diameter using a 3D camera and estimating diameter from the resulting point clouds. Our results indicated that the minimum measurable branch diameter using the Asus Xtion Pro Live is ∼6 mm or less. We also sought to assess the accuracy of the diameter measurements from the 3D camera generated point clouds and how this depended on scanning distance. For branches above 6 mm (for which point cloud data was successfully captured) we found that the accuracy of the device was good from a distance of 50 to 100 cm, and in general measured stem diameters were within a few mm of their true diameter (Figure. 2). However, beyond 100 cm accuracy for all stem diameters declined substantially, suggesting that 3D cameras will need to be used in close proximity to target subjects and that it will be key to maintain an optimal scanning distance to maximize the accuracy of stem geometric reconstructions. Having an indication of the distance of the camera to the object would thus be a valuable option to maintain optimal distance to the object being scanned (50 cm to ∼100 cm by taking into account the proximal blind zone). In general, measurement errors were mostly due to overestimation of diameter, and overestimation was generally consistent (Figure 2). It may thus be possible to calibrate the sensor to diameter measurements to possibly attain better accuracy in diameter measurement. Beyond 1.5 m distance acquisition quality was quite poor. By visually inspecting these acquisitions individually, it was evident that because the depth resolution decreased with distance, the 5 cm gap between the branches and the apparatus support was not detectable at the greater scanning distance.

**Figure 8.**
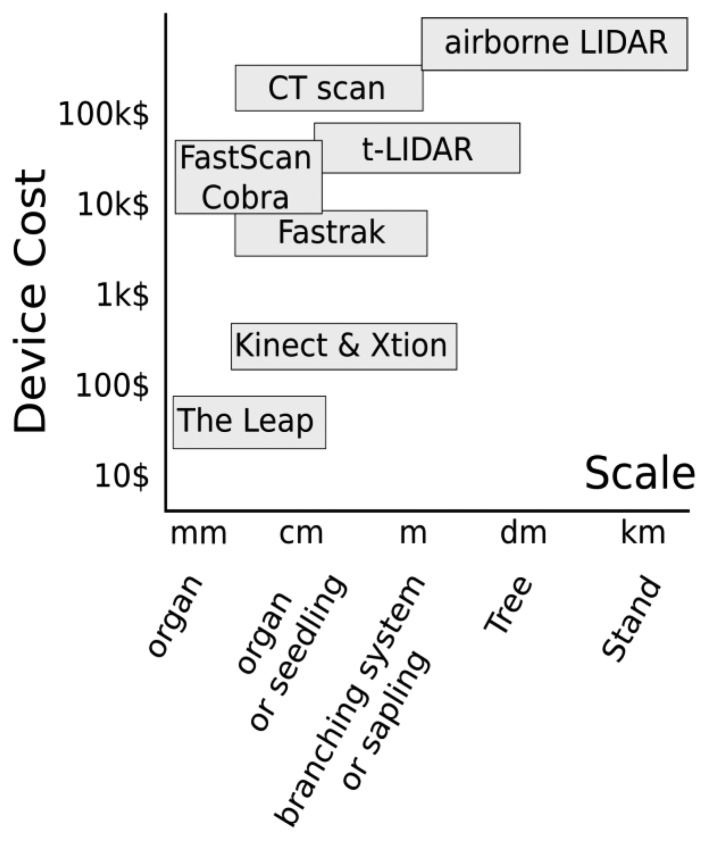
Illustration of devices used to generate 3D architectural data, their prices and the scale at which the generated data is most applicable to woody plants.

The ∼6 mm minimum diameter threshold suggests that many plant stems and the terminal tips of most tree branches (on saplings) could not be measured using the Asus Xtion or similar 3D cameras (Figure 2). However, we note that for saplings and small trees of many species, much of woody aerial structure will be greater than the threshold of detection of the Asus Xtion (or comparable 3D cameras), and that in many cases the threshold of detection may be reasonably similar to TLS devices that are orders of magnitude more costly (Figure 8). In the future, it will be very interesting to see how the technical specifications 3D cameras evolve and how their sensitivity with respect to minimum detectable stem diameters may change. However, we feel researchers should start now with efforts to harness this potential through the development of methods, tools and expertise in order to reap the potential benefits of future technical advancements. In particular, collaboration among plant scientists and computer scientists will be essential, as is the case in the field of FSPM.

Previous work has shown that direct sunlight interferes with the infrared structured light pattern [25], which is likely to be an obstacle for researchers working in bright environments, without a taller plant canopy overhead to intercept IR. This problem is likely to be minimized underneath forest canopies, or alternatively measurements can be made at dawn or dusk. Although measuring at dawn or dusk could be a significant limitation, we think that this will likely not be a large obstacle for the use of 3D cameras in forest environments for two reasons: firstly, forest canopies are often deeply shaded in the understory (although not in gaps). Secondly, next generation technology such as active-IR is reported to allow forthcoming sensors (*i.e.*, Microsoft Kinect sensor to be released with Xbox One or Windows Kinect 2) to work in nearly any lighting condition, although it remains to be determined whether the issue of IR interference by bright light will indeed be solved.

#### 4.1.2. Methods for Obtaining 3D Architectural Data: A Niche for 3D Cameras?

Various devices are available to capture 3D data and there is a large gradient in scale at which data can be generated (Figure 8). 3D magnetic Digitizing (Polhemus Fastrack device) works well for 1 to 10 mm branches and individuals less than 1 m height (seedlings, saplings). Generally, people use a caliper during digitizing to measure diameter and then incorporate it as an attribute associated to segments. For seedlings, measurement time using digitizing is reasonable, requiring somewhere between several minutes to 1 or 2 h depending on crown complexity. As the size and complexity of the target plant increases, digitizing with caliper measurement of diameter becomes less practical and 3D cameras may be more efficient for capturing the majority of the aerial structure of individuals (probably less than 3 m height) since they are capable of detecting 6 mm to at least 50 mm segments. For small to medium sized plants scanning with Xtion from multiple viewpoints to generate point cloud data, may provide a faster alternative than magnetic digitizing. The ratio between measurement time and precision is likely the best for this plant stage and it may be possible to think of measuring a regeneration scene of several individuals as well as leaf on scenes. However, the gain in rapidity of the Xtion will be achieved at the expense of the precision since digitizing records diameter to the closest millimeter.

TLS data is generated by measuring either the time of flight of laser pulses or a change in wavelength frequency after hitting an object. Occlusion of hidden objects, a significant limitation of TLS technologies, is generally solved by performing a series of scans captured from multiple different viewpoints and later registered or by using probability analysis of hit returns [7,12]. TLS will allow scanning scenes of one individual to several large individuals due to its accuracy to detect objects of 1 cm and above. Given that TLS must be several meters away from plants to get valuable point clouds, this places significant constraints (that Xtion does not have) on the use of TLS in forest conditions. For comparable accuracy to TLS, Xtion may thus allow a reduction in occlusion problems by offering more options for (and closer) viewpoints. With 3D scanning and a continuously updated mesh, a new opportunity is presented to be aware during the scanning process of areas of scans with poor point density due to occlusion.

#### 4.1.3. 3D Scanning Using KinFu, ReconstructMe and Skanect

3D scanning, which is based on recently developed methods for the capture of 3D models in real-time, may be able to overcome limited viewing distance of Xtion. Here, we tested a number of software tools available on the web—KinFu, ReconstructMe and Skanect—that perform real-time registration and reconstruction of scenes. This would be desirable when trying to scan from a number of angles to overcome occlusion when scanning small to medium sized plants. In general, continuous 3D scanning performed similarly to static acquisitions with respect to the limits of detection and accuracy of diameter measurements of branches in our simple test (Figures 2 and 4). The increase in the width of the confidence intervals in the continuous scanning acquisition is likely an effect of measurement and data processing, and not likely due to manual measurement in Meshlab. Tuning and optimization of 3D scanning software for the specific measurement of plant structures may be possible, and would potentially increase the sensitivity of diameter detection and increase the utility of the application for measuring plant structures.

By performing 3D scanning of the maple branching system and the cactus, we were able to confirm that the diameter thresholds established in the simple test with the apparatus and vertical branch segments were relevant for 3D scanning of real plants. With respect to time required for plant scanning, the continuous acquisition takes 10 min when the static scanning took 5 min of scanning but 20 min of registration after. Thus continuous 3D scanning is faster for data collection but currently less sensitive. This issue might be overcome with accurate software calibration that allows the retention of more detail. In this study processing power was likely a limitation. For ReconstructMe and Skanect, we found that computer power was a strong limitation on the frame rate and in turn the usability of the devices when high detail settings were used. Since a high performance laptop is required for mobile scanning this will offset the low-cost of 3D cameras relative to other devices somewhat, but not considerably. Battery life might also be an issue as the computer power needed (multicore CPU + CUDA graphic card) is high.

Given that for the optimal resolution plants need to be scanned from ∼1 m distance it is likely that scanning of a small tree (2–3 m tall) would be the maximum potential size one could practically measure. With a 2–3 m tall tree, it is likely that one could still capture much of the crown structure with a strategy of scanning from the outer perimeter of the crown, using either a series of static acquisitions or possibly 3D scanning. With larger specimens, it would likely become necessary to not only scan from the perimeter but also from the interior of the crown. Based on our experience here with the 3D scanning utilities we think that maintaining the continuity of the data collection (*i.e.*, the camera pose relative to the scene) would be very challenging on large trees. During continuous registration scanning, we always included big objects on the ground and focus on them when turning around the plant to ease registration.

#### 4.1.4. Future Research

In this study we were not able to perform a field test of the 3D scanning approach due to the demanding technical requirements for the laptop. However, a field test with a high-performance laptop running some of the software tested here would be an important next step. Leaf area or leaf on scanning would be a logical next step given we have established some limits on the measurement utility for woody structures.

Based on this study and some unpublished work, we suggest that low-cost 3D present interesting alternatives at two scales, both of which might require some specific software development to ease acquisitions. At the organ scale, these devices can be used to get quick measurements of such plant traits as leaf area, stem diameter, and branching pattern used in functional ecology and plant breeding research. Specific development might focus on real-time measurement (shape and colour identification) and numerous repetitions per plants. At the individual scale (or seedling/saplings small scene), 3D cameras can be used to get 3D mock-ups of target individuals for light interception or biomechanical performance evaluation of the plant.

Specific development might focus on real-time registration and occlusion detection in order to provide a high quality point cloud and mesh acquisition of the plant. Indeed, during the continuous acquisitions, we observe some loss of details during the acquisition that might be related to details filtering by the registration algorithm. This filtering is relevant for casual/regular use of continuous registration (e.g., scanning a room or a person) but is an issue for plant architecture scanning.

## 5. Conclusions

Cost effective remote sensing using 3D video cameras is a field of rapid active development, as is the field of 3D modeling of plant architecture. Given the potential for measuring plant woody structure demonstrated here, and the potential for rapid evolution in the capabilities of 3D cameras, plant scientists should be presently working on methods and adapting existing algorithms present in open source libraries to facilitate their use in generating valuable 3D architectural data.

## Figures and Tables

**Table 1.** Specifications and comparison of Asus Xtion Pro Live and Microsoft Kinect.

**Metric**	**Asus Xtion Live Pro**	**Microsoft Kinect**
Price	∼$150 USD	∼$150 USD
Power consumption	<2.5 W	
Distance of use	0.8 m < x < 3.5 m	0.8 m < x < 4 m
Field of view *	58° H; 45° V; 70° D	57.5° H; 43.5° V
Sensor	RGB, Depth and Microphone	RGB, Depth and Microphone
Depth image size	VGA (640 × 480) 30 fps; QVGA (320 × 240) 60 fps	VGA (640 × 480) 30 fps
Resolution	SXVGA (1280*1024)	
Platform	Intel x86; AMD	
OS support	Win 32/64 XP, Vista, 7; Linux Ubuntu 10.10: X86, 32/64 bit, Android	Win 32/64 XP, Vista, 7, 8
Interface	USB2.0	USB2.0
Software	Open NI SDK bundled	Kinect for Windows SDK
Programming languages	C++/C# (Windows); C++ (Linux); Java	C++/C# (Windows); C++ (Linux); Java
Dimensions	18 × 3.5 × 5 cm	28 × 8 × 8 cm

* Horizontal, Vertical and Diagonal.

**Table 2.** Comparison of 3D data capture methods: static acquisition using OpenNI grabber and different software for 3D scanning.

	**Static Acquisition (OpenNI Grabber)**	**3D Scanning Applications**		
		Skanect	KinFu	ReconstructMe
Hardware required	Computer	^a^ Computer	Computer and GPU card with CUDA cores	Computer and GPU card with CUDA cores
Variable detail setting?	No	Yes	No	Yes
Scale optimization		Variable	Room	Variable
Open-source?	Yes	^b^ No	Yes	^c^ No
Measurable dimensions	Yes	Yes	Yes	Yes

Notes:

a Runs faster with GPU card and CUDA cores but possible to use the program without;

b Export of meshes limited for users without a full license.

c Without a full license users must wait 20 s after making an acquisition. See Methods section for software references.
